# The Role of Competition in Structuring Primate Communities under Different Productivity Regimes in the Amazon

**DOI:** 10.1371/journal.pone.0145699

**Published:** 2015-12-22

**Authors:** Juliana Monteiro de Almeida Rocha, Míriam Plaza Pinto, Jean Philippe Boubli, Carlos Eduardo Viveiros Grelle

**Affiliations:** 1 Programa de Pós-Graduação em Ecologia e Conservação da Biodiversidade, Universidade Estadual de Santa Cruz, Ilhéus, Brazil; 2 Departamento de Ecologia, Universidade Federal do Rio Grande do Norte, Natal, Brazil; 3 School of Environment and Life Science, Salford University, Salford, United Kingdom; 4 Departamento de Ecologia, Universidade Federal do Rio de Janeiro, Rio de Janeiro, Brazil; University of Massachusetts Amherst, UNITED STATES

## Abstract

The factors responsible for the formation of Amazonian primate communities are not well understood. Here we investigated the influence of interspecific competition in the assembly of these communities, specifically whether they follow an assembly rule known as "favored states". According to this rule, interspecific competition influences final species composition, resulting in functional groups that are equally represented in the community. We compiled presence-absence data for primate species at 39 Amazonian sites in Brazil, contrasting two regions with distinct productivity regimes: the eutrophic Juruá River basin and the oligotrophic Negro River basin. We tested two hypotheses: that interspecific competition is a mechanism that influences the structure of Amazonian primate communities, and that competition has had a greater influence on the structure of primate communities in regions with low productivity, where resources are more limited. We used null models to test the statistical significance of the results, and found a non-random pattern compatible with the favored states rule in the two regions. Our findings suggest that interspecific competition is an important force driving primate community assembly regardless of productivity regimes.

## Introduction

Assembly rules can be defined as any filter that acts in a regional species pool to determine the structure and composition of species within local communities [1]. Many assembly rules have been proposed, and some of them are well known, e.g. constant body-size ratios [2], guild proportionality [3], species nestedness [4] and trait-environment associations [5]. The structure of local communities can also be influenced by environmental productivity, obscuring local processes such as species interactions [6, 7].

An assembly rule called “favored states”, which is based on functional groups, was firstly proposed by Fox after analyzing small mammal communities in Australia [8]. This rule states that “there is a much higher probability that each species entering a community will be drawn from a different functional group… until each group is represented before the cycle repeats” ([8]: 201). Fox's “functional groups” are equivalent to guilds which we define as groups of species that have similar niches. The rule is based on availability of resources, and the main assumption is that interspecific competition plays an important role in structuring communities. Therefore, each new species that enters a community will tend to be ecologically different from those that are already in the community. When all ecological groups are represented, the rule states that the next species to enter a locality will be from groups which are less well represented in the community. The end result would be that functional groups are equally represented in terms of number of species in a community.

The rule further assumes that stable communities have a higher probability of occurrence [8]. Communities where any possible functional group is absent, or where the number of species among functional groups is unbalanced, will have unexploited resources and consequently will be subject to invasion by species that are able to use this unexploited resource. These communities are considered to be in an "unfavored state", since they have a low probability of occurrence [8]. In contrast, communities where resources are explored more efficiently will have a similar number of species among functional groups. These communities are stable and therefore have a high probability of occurrence in nature, so they are considered to be in a "favored state" [8]. Another assumption of this rule is that resource availability across functional groups is similar. If it is true, the number of species in different functional groups differs by no more than one species in stable communities. When any resource is disproportionately abundant, however, the relationship between groups can be less balanced, because skewed resource availability conditions can support more individuals and species in a particular functional group or groups than in others [8, 9]. In this case, the expected ratio between groups' species richness should be adapted to incorporate such differences [8–10].

Fox and others confirmed this assembly rule for rodents [8–11], and other studies confirmed it for different animal groups, e.g. lemurs [12], shrews [13, 14], and salamanders [15]. Ganzhorn was the first to confirm the rule for arboreal mammals [12]. In addition, although this rule was designed for animal communities, it has also been investigated for plants, with both positive and negative results [16, 17].

Amazonia has the greatest diversity of primates in the world [18]. Although human activity threatens the future and persistence of Amazonian mammals [19,20], some regions of this biome are still pristine and offer an opportunity to study community structure. Grelle [21] analyzed species richness distribution along many sites in the Amazon, and found only one species of primate per genus in each site. This pattern could be the result of interspecific competition reducing the probability of co-occurrence of ecologically similar species [21]. We build off this work to test whether Amazonian primate communities follow the prediction of Fox’s assembly rule. Our initial hypothesis was that interspecific competition is a mechanism that plays a role in structuring these communities.

The Amazon forest is dissected by many large rivers that differ in several physical and chemical aspects, depending on the geology of the areas they drain [22–24]. River type and fluvial dynamics affect soil productivity and therefore influence habitat diversity [25–27]. In this study, we chose localities along a white-water river (Juruá) and a black-water river (Negro). Forests near white-water rivers receive an annual influx of nutrient-rich alluvial sediments [26, 28]. The amount of alluvial sediment can affect the amount of macronutrients available to plants and, consequently, primary productivity [29–31]. Whereas the Negro River sites can be considered nutrient poor, or oligotrophic, the Juruá sites are nutrient rich or eutrophic. It is possible that the structure of communities is influenced by environmental productivity because competition for food is expected to be high when resources are limited [32]. We therefore formulated an additional hypothesis, that competition is more important in determining community structure in regions with low productivity.

## Materials and Methods

**Fig 1:**
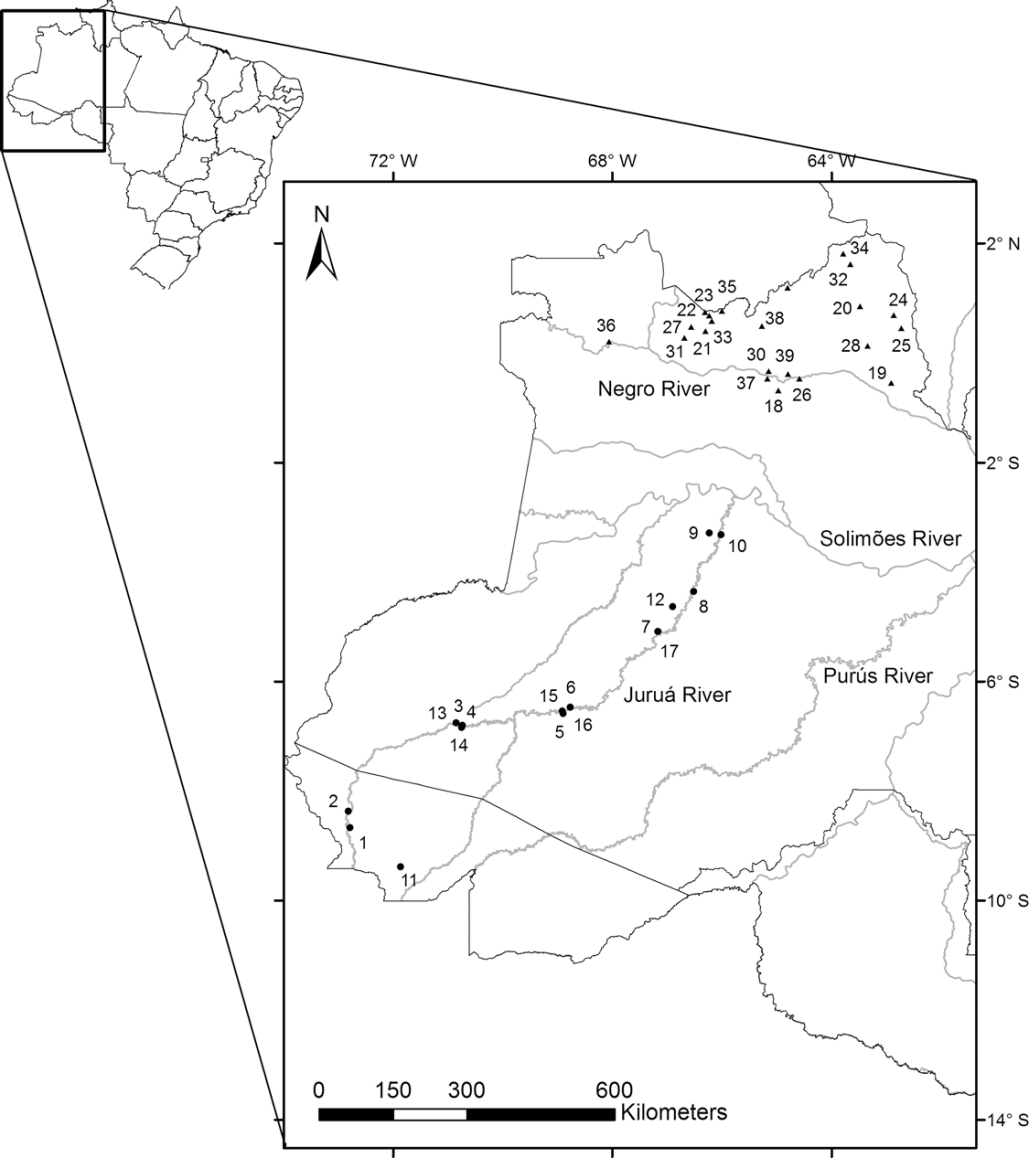
Amazonian localities from which the data were obtained for this study. 1–17: Communities along the Juruá River; 18–39: Communities along the Negro River. For locality names see Table 2.

To test our hypothesis we used published data sets [33–36]. We compiled presence-absence data for primate species from 39 Amazonian sites in Brazil: 17 sites along the Juruá River [33], a high productivity region, and 22 along the Negro River [34–36], a low productivity region (Fig 1). The Juruá is one of the largest white-water tributaries of the Amazon River. Its ‘white’ water appearance is due to the large amount of sediment it carries from its source in the Peruvian Andes [37]. The Negro is the largest northern tributary of the Amazon River and the fifth largest river in the world [38]. This river rises in lowland areas of Colombia, Venezuela and Brazil, and drains a geologically ancient region characterized by impoverished, leached white sandy soils [39–40]. Unlike the Juruá, the Negro is almost completely devoid of sediment [41]. Its black coloration comes from a high concentration of humic acid, a result of incomplete decomposition of leaf litter under the acidic conditions in the white sandy soil forests (caatingas or campinaranas) [42].

The definition of functional groups is crucial for effectively testing this assembly rule [43]. It is necessary to find a balance between categories which are not so broad that groups contain too many different species for competition to remain detectable (the "dilution effect": [44]), and which are not so restricted that groups contain only a few species and potential competitors are underestimated. We defined the functional groups using dietary information available in the literature [27, 45–47]. All neotropical primates include fruits in their diets to different degrees [46]. Some species have omnivorous diets and opportunistically feed on fruits, leaves, insects and small vertebrates in different proportions [46]. Based on such differences, we considered here seven possible functional groups: folivorous/frugivorous (Fo/Fr), frugivorous/folivorous (Fr/Fo), frugivorous/folivorous/granivorous (Fr/Fo/Gr), granivorous/frugivorous (Gr/Fr), frugivorous/insectivorous (Fr/In), frugivorous/omnivorous (Fr/Om) and insectivorous/exudativorous (In/Ex).

We used null models to test Fox’s assembly rule through a randomization procedure. Connor & Simberloff [48] introduced the use of null models in analyses of species co-occurrence to statistically test whether observed patterns are different from what is expected based on chance alone. The idea is to produce a pattern that would be expected in the absence of a particular ecological mechanism, with the aim of verifying whether the pattern observed could be generated at random, or if it is related to a specific structure in the communities [49]. For each real community, we produced random communities by resampling (without replacement) the same number of species observed in the real community from a potential species pool. In this way, the probability of a member of each functional group being drawn is proportional to the number of species in that group in the potential pool.

To construct the random communities, we chose to use the potential species pool instead of drawn species from the observed regional species pool, since the latter may already be a result of past competition. This problem was firstly identified by Cowell & Winkler [50] as the "Narcissus Effect", that corresponds to situations when results of past interactions are included into a model so it cannot be construed as a really null model. Thus, for each locality the potential species pool was drawn up based on maps of potential distribution provided by the IUCN [51] and field data sets [33–36]. We compiled 38 potential species: 27 in the Juruá River region (S1 Table) and 16 in the Negro River region (S2 Table). We were not able to use the same potential pool for all localities in both regions as these differed strongly in some cases (S1 and S2 Tables), mainly in the location of some communities on different riverbanks. Rivers are generally important geographical barriers for these primate species [52–54]. Thus, we built a local potential species pool for each locality including only those species with geographic distributions encompassing the locality. Therefore, we avoided co-occurrences that are not possible in nature and consequently would lead to erroneous conclusions [55].

The 38 species were assigned to one of the seven functional groups (Table 1). The In/Ex group is absent from the Negro River region since no species of this group potentially occur in this region (Table 1). Examining the functional groups, we noted that some changes would be necessary in our approach. It is evident that the Fr/In group contains many more species than the other groups in the Juruá River region (Table 1). Therefore, it is reasonable to expect higher resource availability for this group than for others. This situation violates one of the main assumptions of Fox's assembly rule, that resource availability is the same for all functional groups [8]. In this case, the expected ratios of species richness should be adapted to take this difference into account, as suggested by Fox [8, 9]. To this end we calculated the average number of species observed in each functional group in all communities by region and used these values as indicators of the availability of resources for each functional group. The expected ratio of species in the functional groups Fo/Fr:Fr/Fo:Fr/Fo/Gr:Gr/Fr: Fr/In:Fr/Om:In/Ex was 1:1:1:1:3:2:0 in Juruá River region and remained as 1:1:1:1:1:1 in Negro River region (Table 2). In other words, a community is classified as occupying a favored state when the difference between the number of species in each functional group is equal to or less than that expected based on this ratio, assuming greater resource availability for some groups.

**Table 1:** Classification of primate species with potential occurrence in the two study regions (Juruá and Negro River regions) into functional groups based on dietary preferences. 1 and 0 indicates, respectively, presence and absence of each species in the regions.

species	common name	functional group	Negro river	Juruá river
*Alouatta juara*	Juruá red howler monkey	Fo/Fr	0	1
*Alouatta puruensis*	Purús red howler monkey	Fo/Fr	0	1
*Alouatta macconnelli*	Guianan red howler monkey	Fo/Fr	1	0
*Alouatta seniculus*	Colombian red howler monkey	Fo/Fr	1	0
*Ateles belzebuth*	Red-handed howler monkey	Fr/Fo	1	0
*Ateles chamek*	Black-faced black spider monkey	Fr/Fo	0	1
*Lagothrix cana*	Geoffroy's wolly monkey	Fr/Fo	0	1
*Lagothrix poeppigii*	Poeppig's wolly monkey	Fr/Fo	0	1
*Callicebus cupreus*	Red titi monkey	Fr/Fo/Gr	0	1
*Callicebus purinus*	Red-bellied collared titi monkey	Fr/Fo/Gr	0	1
*Callicebus regulus*	Juruá collared titi monkey	Fr/Fo/Gr	0	1
*Callicebus lugens*	Widow monkey	Fr/Fo/Gr	1	0
*Callicebus torquatus*	White-collared titi monkey	Fr/Fo/Gr	1	0
*Cacajao hosomi*	Neblina uakari	Gr/Fr	1	0
*Cacajao melanocephalus*	Golden-backed black uakari	Gr/Fr	1	0
*Cacajao ayresi*	Ayres' black uakari	Gr/Fr	1	0
*Cacajao calvus*	Bald uakari	Gr/Fr	0	1
*Chiropotes israelita*	Rio negro bearded saki	Gr/Fr	1	0
*Pithecia albicans*	Buffy saki	Gr/Fr	0	1
*Pithecia monachus*	Monk saki	Gr/Fr	0	1
*Pithecia irrorata*	Gray's bald-faced saki	Gr/Fr	0	1
*Saimiri macrodon*	Ecuadorian squirrel monkey	Fr/In	0	1
*Saimiri cassiquiarensis*	Humboldt's squirrel monkey	Fr/In	1	0
*Saimiri boliviensis*	Bolivian squirrel monkey	Fr/In	0	1
*Aotus nigriceps*	Black-headed night monkey	Fr/In	0	1
*Aotus nancymaae*	Nancy Ma's night monkey	Fr/In	0	1
*Aotus vociferans*	Noisy night monkey	Fr/In	1	0
*Aotus trivirgatus*	Northern night monkey	Fr/In	1	0
*Saguinus fuscicollis*	Spix's saddleback tamarin	Fr/In	0	1
*Saguinus imperator*	Emperor tamarin	Fr/In	0	1
*Saguinus melanoleucus*	White saddleback tamarin	Fr/In	0	1
*Saguinus mystax*	Moustached tamarin	Fr/In	0	1
*Saguinus inustus*	Mottled-faced tamarin	Fr/In	1	0
*Callimico goeldii*	Goeldi's monkey	Fr/In	0	1
*Sapajus apella*	Guianan brown tufted capuchin	Fr/Om	1	1
*Cebus olivaceus*	Wedge-capped capuchin	Fr/Om	1	0
*Cebus albifrons*	White-fronted capuchin	Fr/Om	1	1
*Cebuella pygmaea*	Pygmy marmoset	In/Ex	0	1
**total: 38 species**			**16**	**24**

Fo/Fr: folivorous/frugivorous; Fr/Fo: frugivorous-folivorous, Fr/Fo/Gr: frugivorous-folivorous/granivorous; Gr/Fr: granivorous/frugivorous; Fr/In: frugivorous/insectivorous; Fr/Om: frugivorous-omnivorous, and In/Ex: insectivorous-exudativorous.

**Table 2:** Number of species registered in each locality by functional group, mean richness by functional group in each region and ratio calculation.

Locality	Fo/Fr	Fr/Fo	Fr/Fo/Gr	Gr/Fr	Fr/In	Fr/Om	In/ Ex	local richness
Porongaba	1	1	1	1	5	2	1	12
Sobral	1	1	1	2	4	2	0	11
Condor	1	2	1	1	4	2	0	11
Penedo	1	1	1	1	3	2	1	10
Altamira	1	2	1	1	3	2	1	11
Barro Vermelho I	1	2	1	1	4	2	1	12
Fortuna	1	2	2	2	4	2	1	14
Igarapé Jaraqui	1	2	2	1	4	2	1	13
Vira Volta	1	2	2	2	4	2	0	13
Vai Quem Quer	1	1	2	1	4	2	0	11
Reserva Kaxinawá	1	2	1	1	5	2	0	12
Riozinho	1	2	2	2	4	2	1	14
Sacado do Condor	1	0	0	1	1	1	0	4
Nova Empresa	1	0	0	1	2	2	0	6
Boa Esperança	1	1	0	0	1	2	0	5
Barro Vermelho II	1	1	1	1	1	2	0	7
Lago da Fortuna	1	0	1	1	2	2	0	7
**Mean/ratio (Juruá)**	**1.00/1**	**1.29/1**	**1.12/1**	**1.18/1**	**3.24/3**	**1.94/2**	**0.41/0**	
Aiuana	1	0	1	1	3	2	-	8
Araca, Rio	1	0	1	1	1	1	-	5
Araca, Serra	0	1	1	1	1	1	-	5
Bebedor	1	0	1	1	1	1	-	5
Canal Maturaca I	1	1	1	1	1	1	-	6
Canal Maturaca II	1	1	1	1	1	1	-	6
Cuieiras	1	0	1	1	1	0	-	4
Demeni, Cuieiras	1	0	1	1	1	1	-	5
Ecunaui	1	0	1	1	2	2	-	7
Estrada de Maturaca	1	1	1	1	1	1	-	6
Madixi, Igarapé	1	0	1	1	2	1	-	6
Marari	1	1	1	1	0	1	-	5
Marauia, Rio	1	0	1	1	1	1	-	5
Morro Seis Lagos	1	1	1	1	1	1	-	6
Novo Demeni	1	1	1	1	1	1	-	6
Padre, Serra	1	1	1	1	1	1	-	6
Parawa	1	1	1	1	1	1	-	6
Pico Trilha	1	1	1	1	1	1	-	6
uaupes, Ilha Acai	1	0	1	1	3	1	-	7
uneiuxi, serraria	1	0	1	1	3	2	-	8
Xamata	1	1	1	1	1	1	-	6
Daraha. Rio	1	0	1	1	1	1	-	5
**Mean/ratio (Negro)**	**0.95/1**	**0.50/1**	**1.00/1**	**1.00/1**	**1.32/1**	**1.09/1**		

We also noted that the In/Ex group consisted of only one potential species (*Cebuella pygmaea*) in all localities of the Juruá River region, precluding any possibility of competition within this group (Table 1). Taking this group into account, the chance of generating an unfavored state is large even if the other groups are balanced. As a result, a disproportionately large number of communities could be classified as unfavored while at the same time not reflecting the absence of interspecific competition but rather a restriction due to geographical distribution. This problem was identified by Kelt et al. [11], who argue that these communities are less informative. Consequently we did not take this functional group into account for ratio calculations.

The genus *Aotus*, which is part of the Fr/In group, represents the only nocturnal species in the data set (Table 1). It could confound the interpretation of results since *Aotus* spp. can be in greater competition with nocturnal marsupials than with the other diurnal primates. Additionally, no more than one species of this genus occurred at any locality, so we decided to perform two types of models—including and excluding the genus *Aotus* from the communities' classification—to verify if its inclusion could mask a possible pattern compatible with competition only between diurnal species. Finally, considering all modifications in our approach, each locality (real and simulated communities) was classified according to Fox's assembly rule, counting the number of species in each functional group. If species richness was evenly distributed among functional groups, respecting the expected ratios (Table 2), than communities were classified as occupying a "favored state", and if the differences among functional groups' species richness did not follow the expected ratios, they were classified as occupying an "unfavored state".

The simulation procedure was carried out in program R 3.0.2 [56]. We created a function to generate the random communities using the *sample* function from the R Base Package to randomly select species from the potential species pool, and the command *for* to repeat the simulation 10,000 times and generate a distribution of the expected number of favored states over all communities. For example, to generate a random community with five species randomly selected from a set of 11 species (potential species pool: two Fo/Fr, two Fr/Fo, one Fr/Fo/Gr, one Gr/Fr, two Fr/In, two Fr/Om and one In/Ex) 10,000 times, we created the following function:

community >- function(x) {

potential<-{"Fo/Fr1","Fo/Fr2","Fr/Fo1","Fr/Fo2","Fr/Fo/Gr1","Gr/Fr1","Fr/In1",

"Fr/In2","Fr/Om1","Fr/Om2","In/Ex1"}

n<-10,000

random<-5

matrix<-matrix(0,n,random)

for (i in 1:n) {matrix[i,]<-sample(potential,random,replace = FALSE)}

matrix<<-matrix}

The models were run separately for the two regions with and without considering *Aotus* spp. and results were managed in spreadsheets using the software Microsoft Office Excell (2007). After the number of favored states was computed for each null model, statistical significance was determined as the frequency of simulations that had an equal or higher number of favored states than observed communities (α = 0.05).

## Results

Considering only diurnal species, 88% of the Juruá communities (15 out of 17; Table 3) and 81% of the Negro communities (18 out of 22; Table 4) were classified as favored states. When we considered *Aotus* spp. in the ratio calculations, the percentage of communities classified as favored states remained the same in the Juruá River region (Table 3) but decreased to 77% (17 out of 22; Table 4) in the Negro River region.

**Table 3:** Observed species richness by functional group for communities along the Juruá River and classification according to the Fox's assembly rule (F = favored or U = unfavored) with(^1^) and without(^2^) *Aotus* spp.

	Richness by Functional Group								
Expected ratio:	1	1	1	1	3	3	2		
Localities	FO/FR	FR/FO	FR/FO/GR	GR/FR	FR/IN^1^	FR/IN^2^	FR/OM	State^1^	State^2^
1 –Porongaba	1	1	1	1	5	4	2	U	U
2 –Sobral	1	1	1	2	4	3	2	F	F
3 –Condor	1	2	1	1	4	3	2	F	F
4 –Penedo	1	1	1	1	3	2	2	F	F
5 –Altamira	1	2	1	1	3	2	2	F	F
6—Barro Vermelho I	1	2	1	1	4	3	2	F	F
7 –Fortuna	1	2	2	2	4	3	2	F	F
8—Igarapé Jaraqui	1	2	2	1	4	3	2	F	F
9—Vira Volta	1	2	2	2	4	3	2	F	F
10—Vai Quem Quer	1	1	2	1	4	3	2	F	F
11—Reserva Kaxinawá	1	2	1	1	5	4	2	U	U
12 –Riozinho	1	2	2	2	4	3	2	F	F
13—Sacado do Condor	1	0	0	1	1	1	1	F	F
14—Nova Empresa	1	0	0	1	2	1	2	F	F
15—Boa Esperança	1	1	0	0	1	1	2	F	F
16—Barro Vermelho II	1	1	1	1	1	1	2	F	F
17—Lago da Fortuna	1	0	1	1	2	1	2	F	F
**Number of favored states**								**15**	**15**

**Table 4:** Observed species richness by functional group for communities along the Negro River and classification according to the Fox's assembly rule (F = favored or U = unfavored) with(^1^) and without(^2^) *Aotus* spp.

	Richness by Functional Group								
Expected ratio:	1	1	1	1	1	1	1		
Localities	FO/FR	FR/FO	FR/FO/GR	GR/FR	FR/IN^1^	FR/IN^2^	FR/OM	State^1^	State^2^
18-Aiuana	1	0	1	1	3	2	2	U	U
19-Araca, Rio	1	0	1	1	1	0	1	F	F
20-Araca, Serra	0	1	1	1	1	0	1	F	F
21-Bebedor	1	0	1	1	1	0	1	F	F
22-Canal Maturaca I	1	1	1	1	1	0	1	F	F
23-Canal Maturaca II	1	1	1	1	1	0	1	F	F
24-Cuieiras	1	0	1	1	1	1	0	F	F
25-Demeni, Cuieiras	1	0	1	1	1	1	1	F	F
26-Ecunaui	1	0	1	1	2	1	2	U	U
27-Estrada de Maturaca	1	1	1	1	1	0	1	F	F
28-Madixi	1	0	1	1	2	1	1	U	F
29-Marari	1	1	1	1	0	0	1	F	F
30- Marauia	1	0	1	1	1	0	1	F	F
31-Morro Seis Lagos	1	1	1	1	1	0	1	F	F
32-Novo Demeni	1	1	1	1	1	0	1	F	F
33-Padre, Serra	1	1	1	1	1	0	1	F	F
34-Parawa	1	1	1	1	1	1	1	F	F
35-Pico Trilha	1	1	1	1	1	0	1	F	F
36-Uaupes	1	0	1	1	3	2	1	U	U
37-Uneiuxi	1	0	1	1	3	2	2	U	U
38-Xamata	1	1	1	1	1	0	1	F	F
39- Dahara	1	0	1	1	1	1	1	F	F
**Number of favored states**								**17**	**18**

**Fig 2:**
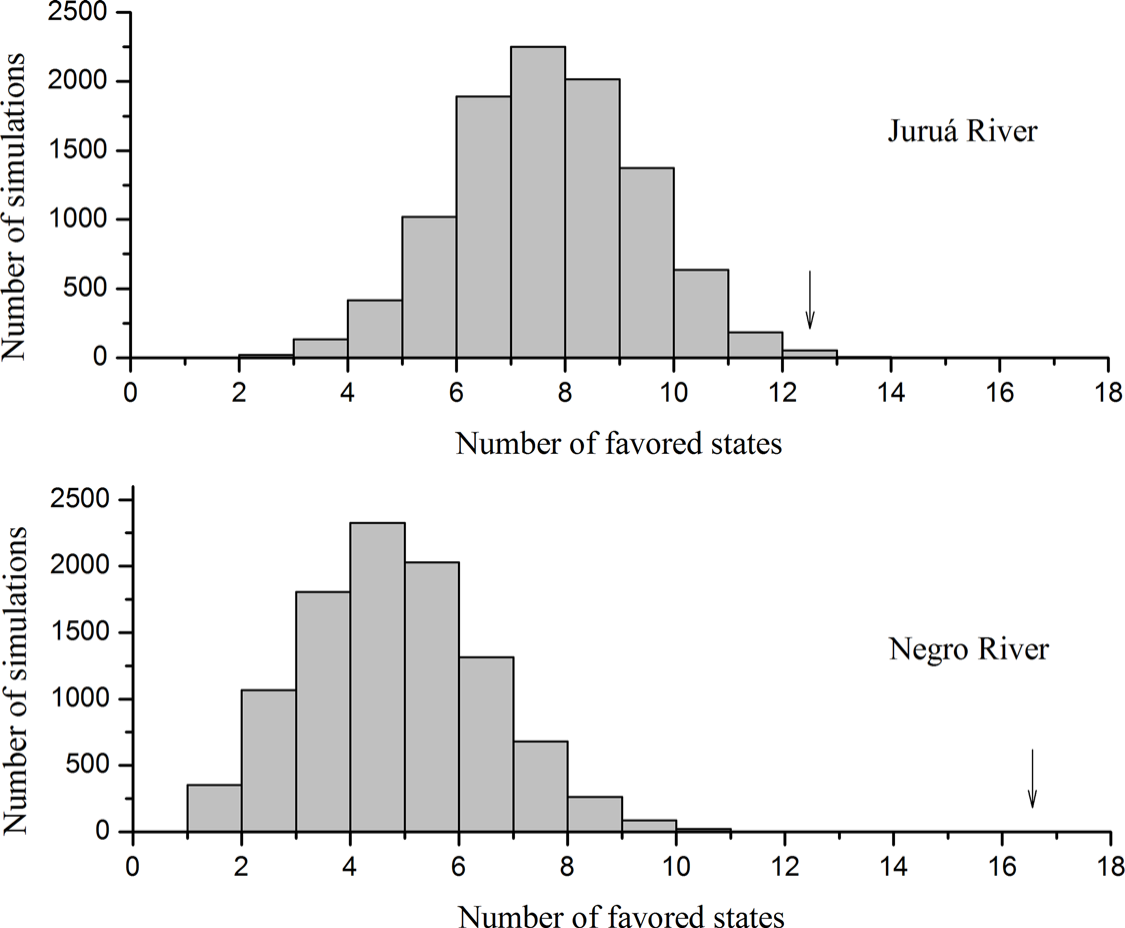
Histograms of null models' simulations considering only diurnal species. Results of null models' simulations with 10,000 randomizations for both regions. The arrows indicate the observed number of communities classified as favored states. We are considering a total of 13 real favored states for the Juruá River region instead of 15 because we did not perform the simulations for the communities Riozinho and Fortuna.

**Fig 3:**
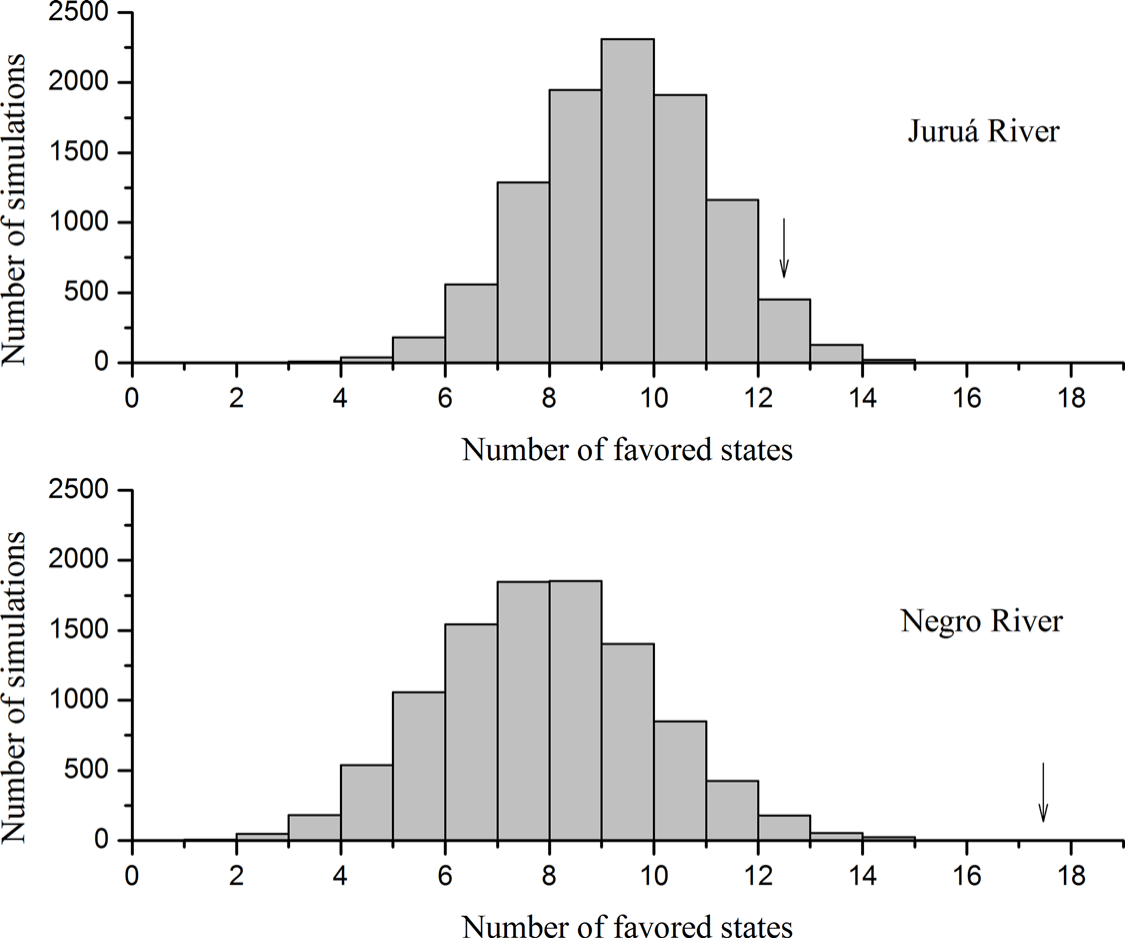
Histograms of null models' simulations considering all species. Results of null models' simulations with 10,000 randomizations for both regions. The arrows indicate the observed number of communities classified as favored states. We are considering a total of 13 real favored states for the Juruá River region instead of 15 because we did not perform the simulations for the communities Riozinho and Fortuna.

In the null model that considered only diurnal species, a mean of eight favored states was generated by simulations in the Juruá River region, and 145 simulations had an equal or higher number of favored states than observed (P = 0.0015; Fig 2). In the second model, including *Aotus* spp., the mean number of favored states decreased to seven and only five simulations had an equal number of favored states than observed (P = 0.0005; Fig 3). Null model simulations were not run for two Juruá localities (Fortuna and Riozinho) because the number of species in the potential and actual species pools were identical, so the result was predictable.

In the Negro River region, we found a mean of seven favored states considering only the diurnal species, and no simulation had an equal or higher number of favored states than was observed in actual communities (P = 0.0000; Fig 2). When we included *Aotus* spp., the mean favored states decreased to four but the number of simulations remained equal to zero (P = 0.0000; Fig 3).

In sum, our results corroborate our first hypothesis, since a pattern compatible with competition was found for the two regions investigated. However, our second hypothesis was not supported as competition was not more important under low productivity regimes. The inclusion of the nocturnal species from the genus *Aotus* in the null models lead to a decrease in the mean number of favored states generated by simulations but did not change the significance of the models.

## Discussion

In this study, we investigated the role of interspecific competition in the community assembly of primates in Amazonia, contrasting regions with different productivity regimes. Our findings support the hypothesis that competition is a primary force driving primate community assembly, regardless of primary productivity. For the two regions investigated here, species coexistence can be driven by competition in a manner proposed by Fox [8].

Schreier et al. [57] noted that demonstrating the role of competition in structuring communities is a difficult task, especially for primates, whose responses to competition might be delayed over decades [58], and suggested that null models provide a means of testing for competition. We attempted to incorporate specific characteristics of our data set in the null models to ensure that we were building plausible models. For example, we built random communities that could exist in these regions, respecting the dispersal limitations imposed by rivers [36, 52]. We also changed the original expected ratio of species richness for the Juruá River region to incorporate the unbalanced availability of resources to the functional groups. We believe these modifications really improved our findings and we strongly encourage authors to consider it when doing this type of analysis.

The main difficult to test this assembly rule is assigning species to broad diet categories. This is a crucial point for the analysis since we have to group species that are really potential competitors otherwise the results would be compromised. If we build functional groups with species that could not compete on nature, we could mask a pattern generated by competition. Otherwise, if we are too restrictive considering every detail in the diet of the species, we could end with one species by group and no possibility for competition. As potential competitors, species have to overlap substantially (but not completely) in their diets. For example, there is no species in the studied regions that feeds on gum with the same degree that *Cebuella pygmaea* does [59], so it is unlikely that this species competes strongly with any other primate species in these communities. In this way, *C*. *pygmaea* is the only species in the In/Ex group, which was not considered for the analysis since do not include potential competitors. We believe that species dietary are well represented in our functional groups since we were very cautious when clustering species based on published dietary information [27, 45–47].

Communities along the Juruá River are more diverse than those along the Negro River so we expected that the higher availability of food resources, resultant of the higher primary productivity in this region, could allow the co-occurrence of more species without competitive exclusion [60]. In fact, we found that communities follow a pattern that can be resultant of competition even if the resource availability is high. Other authors also found different patterns regarding the role of competition in structuring primate communities [12, 57, 61, 62]. For example, Kamilar & Ledogar [61] examined the co-occurrence patterns of primate species at several localities on the three continents where primates occur (Asia, Africa and America). They looked for "checkerboard distributions", a distributional pattern where pairs of species never co-occur due to competitive exclusion [63]. When they analyzed all species without accounting for dietary characteristics, they found a substantial number of checkerboard distributions in all regions except for the region they designated the Central Amazon. This region coincides with the localities we analyzed, including both white and black-water rivers. When these researchers analyzed dietary guilds separately, they found a non-random pattern only for frugivores and insectivores, while for folivore and frugivore-insectivore guilds the pattern was random in almost all regions. Their findings suggest that species with a broader diet are less affected by competition. These guilds might be more influenced by bottom-up processes than by competition.

The majority of papers that investigate competition in primate communities do not include non-primate competitors [57], with the exception of Beaudrot et al. [62], who also searched for checkerboard distributions and found that evidence for competition among primates and other taxa was stronger than for competition between primates only. Although this is a very interesting finding, the authors cautioned that the low diversity of primates in the study region (Borneo) probably implies a reduced number of interactions between primates. They also tested other assembly rules and found support for guild proportionality [3] but not for Fox's assembly rule. However, they argued that their guild categorization may not have been refined enough to detect competition and by extension Fox’s rule. It may be of interest to reanalyze their dataset using a more appropriate guild categorization. In the same way, future investigations of competition in primate communities, especially species-rich communities, should include non-primate competitors where possible. This would help us to better understand the role of competition in primates.

It is worth noting that Fox’s assembly rule was developed for low-diversity communities varying from two to seven species [8]. Fox and Brown [10] cautioned that the rule may need to be modified for more diverse communities. However, Ganzhorn [12] applied the same approach and confirmed the rule's validity for localities with up to 13 species of lemurs. We therefore applied the same approach to our data even though communities along the Juruá River contain up to 14 species. Our results provide evidence for operation of the favored states rule in primate communities in the Amazon. The most interesting finding is that the pattern was consistent between regions with different productivity regimes, suggesting that interspecific competition may be a strong force structuring primate communities.

## Supporting Information

S1 Table Juruá River's potential species pool. Primate species with potential occurrence in 17 localities along the Juruá River.
(DOCX)

S2 Table Negro River's potential species pool. Primate species with potential occurrence in 22 localities along the Negro River.
(DOCX)
