# Supplementary material for: The Role of Competition in Structuring Primate Communities under Different Productivity Regimes in the Amazon
Source: PLoS One. 2015 Dec 22;10(12):e0145699. doi: 10.1371/journal.pone.0145699 (PMC4687872; doi:10.1371/journal.pone.0145699)
Supplement: S1 Table — Primate species with potential occurrence in 17 localities along the Juruá River. (DOCX) [file pone.0145699.s001.docx]

**Table S1. Juruá River's potential species pool.** Localities names and its geographic coordinates are presented below the table.

| **Species** | **Localities** | | | | | | | | | | | | | | | | |
| --- | --- | --- | --- | --- | --- | --- | --- | --- | --- | --- | --- | --- | --- | --- | --- | --- | --- |
|  | **1** | **2** | **3** | **4** | **5** | **6** | **7** | **8** | **9** | **10** | **11** | **12** | **13** | **14** | **15** | **16** | **17** |
| *Phitecia albicans* | 0 | 0 | 0 | 0 | 0 | 0 | 0 | 1 | 0 | 0 | 0 | 0 | 0 | 0 | 0 | 0 | 0 |
| *Pithecia monachus* | 0 | 1 | 1 | 0 | 0 | 1 | 1 | 0 | 1 | 0 | 0 | 1 | 0 | 1 | 0 | 1 | 1 |
| *Pithecia irrorata* | 1 | 0 | 0 | 1 | 1 | 0 | 0 | 1 | 0 | 1 | 1 | 0 | 1 | 0 | 1 | 0 | 0 |
| *Cacajao calvus* | 0 | 1 | 0 | 0 | 0 | 0 | 1 | 0 | 1 | 0 | 0 | 1 | 0 | 0 | 0 | 0 | 1 |
| *Alouatta juara* | 0 | 1 | 1 | 0 | 0 | 1 | 1 | 0 | 1 | 0 | 0 | 1 | 0 | 1 | 0 | 1 | 1 |
| *Alouatta puruensis* | 1 | 0 | 0 | 1 | 1 | 0 | 0 | 1 | 0 | 1 | 1 | 0 | 1 | 0 | 1 | 0 | 0 |
| *Ateles chamek* | 1 | 1 | 1 | 1 | 1 | 1 | 1 | 1 | 1 | 1 | 1 | 1 | 1 | 1 | 1 | 1 | 1 |
| *Lagothrix cana* | 0 | 1 | 1 | 0 | 0 | 1 | 1 | 0 | 1 | 0 | 0 | 1 | 0 | 1 | 0 | 1 | 1 |
| *Lagothrix poepiggi* | 1 | 0 | 0 | 1 | 1 | 0 | 0 | 1 | 0 | 1 | 1 | 0 | 1 | 0 | 1 | 0 | 0 |
| *Callicebus cupreus* | 1 | 1 | 1 | 1 | 1 | 1 | 1 | 1 | 1 | 1 | 1 | 1 | 1 | 1 | 1 | 1 | 1 |
| *Callicebus purinus* | 0 | 0 | 0 | 0 | 0 | 0 | 0 | 1 | 0 | 1 | 0 | 0 | 0 | 0 | 0 | 0 | 0 |
| *Callicebus regulus* | 0 | 0 | 1 | 0 | 0 | 1 | 1 | 0 | 1 | 0 | 0 | 1 | 0 | 1 | 0 | 1 | 1 |
| *Sapajus apella* | 1 | 1 | 1 | 1 | 1 | 1 | 1 | 1 | 1 | 1 | 1 | 1 | 1 | 1 | 1 | 1 | 1 |
| *Cebus albifrons* | 1 | 1 | 1 | 1 | 1 | 1 | 1 | 1 | 1 | 1 | 1 | 1 | 1 | 1 | 1 | 1 | 1 |
| *Saimiri boliviensis* | 1 | 0 | 0 | 1 | 1 | 0 | 0 | 1 | 0 | 1 | 1 | 0 | 1 | 0 | 1 | 0 | 0 |
| *Saimiri macrodon* | 0 | 1 | 1 | 0 | 0 | 1 | 1 | 0 | 1 | 0 | 0 | 1 | 0 | 1 | 0 | 1 | 1 |
| *Callimico goeldii* | 1 | 0 | 0 | 0 | 0 | 0 | 0 | 0 | 0 | 0 | 1 | 0 | 0 | 0 | 0 | 0 | 0 |
| *Cebuella pygmaea* | 1 | 1 | 1 | 1 | 1 | 1 | 1 | 1 | 1 | 1 | 1 | 1 | 1 | 1 | 1 | 1 | 1 |
| *Saguinus fuscicollis* | 1 | 1 | 1 | 1 | 1 | 1 | 1 | 1 | 1 | 1 | 1 | 1 | 1 | 1 | 1 | 1 | 1 |
| *Saguinus imperator* | 1 | 0 | 0 | 0 | 0 | 0 | 0 | 0 | 0 | 0 | 1 | 0 | 0 | 0 | 0 | 0 | 0 |
| *Saguinus melanoleucus* | 1 | 0 | 0 | 1 | 1 | 0 | 0 | 1 | 0 | 1 | 1 | 0 | 1 | 0 | 1 | 0 | 0 |
| *Saguinus mystax* | 1 | 1 | 1 | 1 | 1 | 1 | 1 | 1 | 1 | 1 | 0 | 1 | 1 | 1 | 1 | 1 | 1 |
| *Aotus nigriceps* | 1 | 0 | 0 | 1 | 1 | 0 | 0 | 1 | 0 | 1 | 1 | 0 | 1 | 0 | 1 | 0 | 0 |
| *Aotus nancymaae* | 0 | 1 | 1 | 0 | 0 | 1 | 1 | 0 | 1 | 0 | 0 | 1 | 0 | 1 | 0 | 1 | 1 |
| **Total of species** | **15** | **13** | **13** | **13** | **13** | **13** | **14** | **15** | **14** | **14** | **14** | **14** | **13** | **13** | **13** | **13** | **14** |

Note: Potential species pool were designed based on maps of potential distribution provided by IUCN (2014) and data sets (Peres 1997).

Localities names and its coordinates (datum: SAD 69)

| 1 | Porongaba | -72.78231 | -8.66591 |
| --- | --- | --- | --- |
| 2 | Sobral | -72.81571 | -8.36591 |
| 3 | Condor | -70.84902 | -6.74921 |
| 4 | Penedo | -70.74902 | -6.83251 |
| 5 | Altamira | -68.89902 | -6.58251 |
| 6 | Barro Vermelho I | -68.76572 | -6.46591 |
| 7 | Fortuna | -67.16573 | -5.08251 |
| 8 | Igarapé Jaraqui | -66.51573 | -4.34922 |
| 9 | Vira Volta | -66.23233 | -3.28252 |
| 10 | Vai Quem Quer | -66.01574 | -3.31592 |
| 11 | Reserva Kaxinawá | -71.86571 | -9.38251 |
| 12 | Riozinho | -66.89903 | -4.63252 |
| 13 | Sacado do Condor | -70.84902 | -6.74921 |
| 14 | Nova Empresa | -70.73232 | -6.79921 |
| 15 | Boa Esperança | -68.91572 | -6.53251 |
| 16 | Barro Vermelho II | -68.76572 | -6.46591 |
| 17 | Lago da Fortuna | -67.16573 | -5.08251 |
