# Supplementary material for: The Role of Competition in Structuring Primate Communities under Different Productivity Regimes in the Amazon
Source: PLoS One. 2015 Dec 22;10(12):e0145699. doi: 10.1371/journal.pone.0145699 (PMC4687872; doi:10.1371/journal.pone.0145699)
Supplement: S2 Table — Primate species with potential occurrence in 22 localities along the Negro River. (DOCX) [file pone.0145699.s002.docx]

**Table S2. Negro River's potential species pool.** Localities names and its geographic coordinates are presented below the table.

| **Species** | **Localities** | | | | | | | | | | | | | | | | | | | | | |
| --- | --- | --- | --- | --- | --- | --- | --- | --- | --- | --- | --- | --- | --- | --- | --- | --- | --- | --- | --- | --- | --- | --- |
|  | **18** | **19** | **20** | **21** | **22** | **23** | **24** | **25** | **26** | **27** | **28** | **29** | **30** | **31** | **32** | **33** | **34** | **35** | **36** | **37** | **38** | **39** |
| *Cacajao hosomi* | 0 | 0 | 0 | 1 | 1 | 1 | 0 | 0 | 0 | 1 | 0 | 0 | 0 | 1 | 0 | 1 | 0 | 1 | 0 | 0 | 1 | 0 |
| *Cacajao melanocephalus* | 1 | 1 | 0 | 1 | 1 | 1 | 0 | 0 | 1 | 1 | 1 | 1 | 1 | 1 | 1 | 1 | 1 | 1 | 1 | 1 | 1 | 1 |
| *Cacajao ayresi* | 0 | 1 | 0 | 0 | 0 | 0 | 0 | 0 | 0 | 0 | 1 | 0 | 0 | 0 | 0 | 0 | 0 | 0 | 0 | 0 | 0 | 0 |
| *Chiropotes israelita* | 0 | 1 | 1 | 1 | 1 | 1 | 1 | 1 | 0 | 1 | 1 | 1 | 1 | 1 | 1 | 1 | 1 | 1 | 0 | 0 | 1 | 1 |
| *Alouatta maconelli* | 0 | 1 | 1 | 1 | 1 | 1 | 1 | 1 | 0 | 1 | 1 | 1 | 1 | 1 | 1 | 1 | 1 | 1 | 0 | 0 | 1 | 1 |
| *Alouatta seniculus* | 1 | 0 | 0 | 0 | 0 | 0 | 0 | 0 | 1 | 0 | 0 | 0 | 0 | 0 | 0 | 0 | 0 | 0 | 1 | 1 | 0 | 0 |
| *Ateles belzebuth* | 1 | 1 | 1 | 1 | 1 | 1 | 1 | 1 | 1 | 1 | 1 | 1 | 1 | 1 | 1 | 1 | 1 | 1 | 1 | 1 | 1 | 1 |
| *Callicebus lugens* | 0 | 1 | 1 | 1 | 1 | 1 | 1 | 1 | 0 | 1 | 1 | 1 | 1 | 1 | 1 | 1 | 1 | 1 | 1 | 0 | 1 | 1 |
| *Callicebus torquatus* | 1 | 0 | 0 | 0 | 0 | 0 | 0 | 0 | 1 | 0 | 0 | 0 | 0 | 0 | 0 | 0 | 0 | 0 | 0 | 1 | 0 | 0 |
| *Sapajus apella* | 1 | 1 | 1 | 1 | 1 | 1 | 1 | 1 | 1 | 1 | 1 | 1 | 1 | 1 | 1 | 1 | 1 | 1 | 1 | 1 | 1 | 1 |
| *Sapajus olivaceus* | 0 | 1 | 1 | 0 | 0 | 0 | 0 | 1 | 0 | 0 | 1 | 0 | 0 | 0 | 0 | 0 | 0 | 0 | 0 | 0 | 0 | 0 |
| *Cebus albifrons* | 1 | 1 | 1 | 1 | 1 | 1 | 1 | 1 | 1 | 1 | 1 | 1 | 1 | 1 | 1 | 1 | 1 | 1 | 1 | 1 | 1 | 1 |
| *Aotus trivirgatus* | 0 | 1 | 1 | 1 | 1 | 1 | 1 | 1 | 0 | 1 | 1 | 1 | 1 | 1 | 1 | 1 | 1 | 1 | 0 | 0 | 1 | 1 |
| *Aotus vociferans* | 1 | 0 | 0 | 0 | 0 | 0 | 0 | 0 | 1 | 0 | 0 | 0 | 1 | 0 | 0 | 0 | 0 | 0 | 1 | 1 | 0 | 1 |
| *Saguinus inustus* | 1 | 0 | 0 | 0 | 0 | 0 | 0 | 0 | 1 | 0 | 0 | 0 | 0 | 0 | 0 | 0 | 0 | 0 | 1 | 0 | 0 | 0 |
| *Saimiri cassiquiarensis* | 1 | 1 | 1 | 1 | 1 | 1 | 1 | 1 | 1 | 1 | 1 | 1 | 1 | 1 | 1 | 1 | 1 | 1 | 1 | 1 | 1 | 1 |
| **Total of species** | **9** | **11** | **9** | **10** | **10** | **10** | **8** | **9** | **9** | **10** | **11** | **9** | **10** | **10** | **9** | **10** | **9** | **10** | **9** | **8** | **10** | **10** |

Note: Potential species pool were designed based on maps of potential distribution provided by IUCN (2014) and data sets (Boubli 1997; Boubli *et al.* 2008; Boubli *et al*. 2015).

Localities names and its coordinates (datum: SAD 69)

| 18 | Aiuana | -64.96794 | -0.68923 |
| --- | --- | --- | --- |
| 19 | Araca, Rio | -62.91025 | -0.54733 |
| 20 | Araca, Serra | -63.48045 | 0.85297 |
| 21 | Bebedor | -66.29874 | 0.39937 |
| 22 | Canal Maturaca I | -66.30984 | 0.74447 |
| 23 | Canal Maturaca II | -66.22914 | 0.68217 |
| 24 | Cuieiras | -62.86345 | 0.69247 |
| 25 | Demeni, Cuieiras | -62.72305 | 0.45307 |
| 26 | Ecunaui | -64.58764 | -0.47073 |
| 27 | Estrada de Maturaca | -66.56003 | 0.47387 |
| 28 | Madixi, Igarapé | -63.33705 | 0.12607 |
| 29 | Marari | -64.80074 | 1.18637 |
| 30 | Marauia, Rio | -65.14904 | -0.33253 |
| 31 | Morro Seis Lagos | -66.68083 | 0.27477 |
| 32 | Novo Demeni | -63.65285 | 1.61616 |
| 33 | Padre, Serra | -66.18404 | 0.58397 |
| 34 | Parawa | -63.78344 | 1.80746 |
| 35 | Pico Trilha | -65.99704 | 0.76347 |
| 36 | uaupes, Ilha Acai | -68.05523 | 0.20247 |
| 37 | uneiuxi, serraria | -65.16964 | -0.47193 |
| 38 | Xamata | -65.27074 | 0.49247 |
| 39 | Daraha. Rio | -64.78904 | -0.38923 |
